# Supplementary material for: Innate Resistance and Phosphite Treatment Affect Both the Pathogen’s and Host’s Transcriptomes in the Tanoak-Phytophthora ramorum Pathosystem
Source: J Fungi (Basel). 2021 Mar 9;7(3):198. doi: 10.3390/jof7030198 (PMC7999100; doi:10.3390/jof7030198)
Supplement: Supplementary file 1 [file jof-07-00198-s001.zip › Supple_Deg_2020/DocumentS1_2020v1.docx]

Document S1

Fisher’s exact test and Wilcox test were performed on R as follows.

# 3. RESULTS

# 3.2. Clustering of expression revealed factors influencing tanoak transcriptomes

## Cluster A was enriched with T7 samples with susceptible phenotypes

|  | A | B+C | total |
| --- | --- | --- | --- |
| S | 4 | 3 | 7 |
| R + N | 1 | 12 | 13 |

10/29/2019, 11/15/2020

fisher.test(rbind(c(4,3),c(1,12)))

Fisher's Exact Test for Count Data

data: rbind(c(4, 3), c(1, 12))

p-value = 0.0307

## Cluster C is enriched with uninfected samples (all T0 samples and non-inoculated T7 samples, p=3.0x10^-5^).

|  | A+B | C | total |
| --- | --- | --- | --- |
| Uninfected | 0 | 15+2 | 17 |
| infected | 12 | 6 | 18 |

10/29/2019, 11/15/2020

fisher.test(rbind(c(17,0),c(6,12)))

Fisher's Exact Test for Count Data

data: rbind(c(17, 0), c(6, 12))

p-value = 2.966e-05

# 3.3. Search for tanoak transcriptome signatures associated with innate resistance and phosphite treatment

## The number of sequences mapped at T7 did not correlated with the observed disease responses, which were scored at 35 DPI (Fig. 3). Mapped gene models >50,000

|  | P. ramorum + | P. ramorum - | total |
| --- | --- | --- | --- |
| R | 3 | 9 | 12 |
| S | 4 | 3 | 7 |

5/30/2019, 11/27/2019, 11/15/2020

> fisher.test(rbind(c(3,9),c(4,3)))

Fisher's Exact Test for Count Data

data: rbind(c(3, 9), c(4, 3))

p-value = 0.2711

## Although phosphite-untreated susceptible plants showed high level of P. ramorum reads, difference between phosphite-treated and untreated samples was not statistically significant (Mann Whitney U test, p=0.095, Document S1).

| fileName | TP | PR |
| --- | --- | --- |
| HS1A_index3 | CS | 2713332.00 |
| HS3A_index16 | CS | 1756127.00 |
| HS4B_index9 | CS | 1031399.00 |
| HS1A_index11 | CS | 239762.00 |
| HS1A_index4 | CS | 30409.00 |
| HS1A_index8 | PS | 20207.00 |
| HS1A_index5 | PS | 11165.00 |

1/10/2020, 11/15/2020

**Cs>Ps**

**Mann Whitney U Test = Wilcoxon Ran Sum Test**

Is Reads in Cs more than those in Ps?

prTable<-read.clipboard()

wilcox.test(prTable$PR ~prTable$TP, data=prTable)

Wilcoxon rank sum test

W = 10, p-value = 0.09524

## All seven samples with detectable level of *P. ramorum* transcripts were found in either Cluster A or B (p=4.0x10^-3^)

|  | P. ramorum + | P. ramorum - | total |
| --- | --- | --- | --- |
| A+B | 7 | 3 | 10 |
| Not A or B | 0 | 8 | 8 |

11/11/2020

fisher.test(rbind(c(7,3),c(0,8)))

Fisher's Exact Test for Count Data

data: rbind(c(7, 3), c(0, 8))

p-value = 0.004022

## Global mRNA expression pattern of tanoak at T7 was most strongly associated with active growth of the pathogen inside the host tissue, but not with the disease phenotypes at T35 (*p*=0.27) or the phosphite treatment (*p*=0.37)

| Inoculated T7 samples | Phi + | control | Total |
| --- | --- | --- | --- |
| A+B | 3 | 7 | 10 |
| not A or B | 6 | 4 | 10 |

6/16/2018, 11/16/2020

fisher.test(rbind(c(3,7),c(6,4)))

Fisher's Exact Test for Count Data

data: rbind(c(3, 7), c(6, 4))

p-value = 0.3698
